# Investigation of topical amniotic membrane suspension and ReGeneraTing Agent on early corneal stromal healing in rats

**DOI:** 10.1016/j.vas.2024.100344

**Published:** 2024-03-07

**Authors:** Hao Lee, Wei-Hsiang Huang, Yi-Chen Sun, Chung-Tien Lin

**Affiliations:** a Institute of Veterinary Clinical Sciences, School of Veterinary Medicine, National Taiwan University, Taipei, Taiwan; b Department of Ophthalmology, National Taiwan University Veterinary Hospital, Taipei, Taiwan; c Graduate Institute of Molecular and Comparative Pathobiology, School of Veterinary Medicine, National Taiwan University, Taipei, Taiwan; d Department of Ophthalmology, Taipei Tzu Chi General Hospital, New Taipei City, Taiwan

**Keywords:** Amniotic membrane suspension, Corneal ulcer, Corneal healing, ReGeneraTing Agent, Optical coherence tomography

## Abstract

Innovative topical bioregenerative materials promoting corneal stromal healing provide valuable alternatives for treating patients with deep corneal ulcers, and particularly beneficial for those with a higher anaesthetic risk. This study aimed to investigate the effects of topical amniotic membrane suspension (AMS) and ReGeneraTing Agent (RGTA) on surgically induced deep stromal ulcers in rats. Eighteen Sprague-Dawley rats were divided into 3 treatment groups: control group (topical normal saline, TID); AMS group (topical AMS, TID); RGTA group (topical RGTA, Q2D). Corneal microsurgery was used to create deep stromal ulcer. Evaluations were performed by corneal opacity grading, spectral domain optical coherence tomography (SD-OCT), histopathology, and immunohistochemistry. One-way ANOVA and Dunnett's test were used for statistical analysis. By the seventh day of treatment, both the AMS and RGTA groups showed significantly greater thickness in corneal stroma (both *p*-value < 0.05) than the control group. Additionally, the RGTA group exhibited a significantly higher degree of myofibroblast infiltration in the stroma and a greater level of corneal opacity (*p* < 0.05). No significant differences in the count of inflammatory cells were noted. In conclusion, both AMS and RGTA have demonstrated effectiveness in promoting the early stages of stromal wound healing and wound defect recovery in our research. Both AMS and RGTA have good potential for treating deep corneal ulcers in small animals practice. Further research is necessary to investigate the long-term effects and mechanism of using topical AMS and RGTA on treating deep corneal ulcer in clinical practice.

## Highlights

• Amniotic Membrane Suspension (AMS) and ReGeneraTing Agent (RGTA) have been shown to enhance the early stages of corneal stromal healing.
• The enhancement in corneal wound healing was evident from the increased corneal stromal thickness as observed under optical coherence tomography (OCT) examination.
• Further research is necessary to investigate the long-term effects and mechanism of using topical AMS and RGTA on treating deep corneal ulcer in clinical practice.

## Graphical abstract

**Figure:**
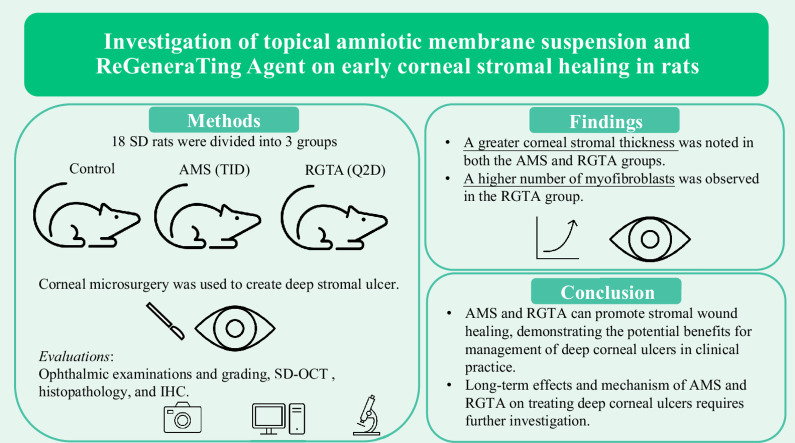

## 1 Introduction

In veterinary eye care, deep corneal ulcers present a critical challenge, often leading to severe discomfort and potential vision impairment. The surgical repair is typically essential to promote healing of the corneal stroma and ensure the integrity of the cornea's structure (Gelatt et al., 2021). However, for patients with underlying systemic health issues or advanced age, the heightened risks tied to anesthesia may render surgery an impractical choice. Thus, the development and implementation of a non-surgical medical treatment that can effectively foster the corneal stromal healing process is of considerable importance.

The amniotic membrane has long played a pivotal role in human corneal surgery (Meller et al., 2011), contributing to both the structural fortification of the cornea and the acceleration of the healing process. This is due to its ability to preserve the biochemical properties of the corneal material while delivering essential extracellular matrix components to the damaged cornea (Guo et al., 2011; Shimmura & Tsubota, 2002). Additionally, amniotic membrane exhibits antifibrotic and antiangiogenic effects, mitigating corneal scarring and maintaining transparency (Shimmura & Tsubota, 2002). The topical amniotic membrane suspension (AMS) is a new product (EyeQ Amniotic Eye DropsⓇ, Vetrix, Cumming, GA, USA) for use in veterinary ophthalmology in recent years. To the best of our knowledge, there is a lack of controlled studies investigating the efficacy of amniotic membrane suspension in an animal model specifically designed to evaluate deep stromal ulcers.

ReGeneraTing Agents (RGTA) are synthetic polymers designed to emulate and replace the function of heparan sulfate in corneal tissue injuries (Chebbi et al., 2008). Serving as a substitute for the extracellular matrix, RGTAs bear both structural and functional resemblances to the naturally occurring heparan sulfate found within corneal tissue (Chebbi et al., 2008). A significant feature of RGTAs is their resilience to enzymatic breakdown, which grants them the stability to persist within the challenging environment of an injured cornea (Barritault et al., 2017). Optixcare Eye EMSⓇ (Optixcare Eye EMSⓇ, Aventix, Ontario, Canada) represent a novel extracellular matrix substitute product of RGTA for use in veterinary ophthalmology. Nevertheless, to the best of our knowledge, no published research has yet examined the therapeutic effect of RGTA on deep stromal ulcer.

The purpose of the study was to assess the effects of commercially available AMS (EyeQ Amniotic Eye DropsⓇ) and RGTA (Optixcare Eye EMSⓇ) in a rat model of deep corneal stromal ulcer.

## 2 Material and methods

### 2.1 Experimental animals and groups

The experiments and use of animals in the study were approved by the Institutional Animal Care and Use Committee (IACUC Approval No.: NTU-110-EL-00082, issued on July 28, 2021) of the first author's institution for the procedures and the care of animals. Eighteen female Sprague-Dawley rats were kept under controlled environmental conditions (20–25 ℃, 12 h light / dark cycle) for at least 3 weeks before starting the experiments. By the time surgical procedures were initiated, the rats were between 6 and 8 weeks old and weighed between 180 and 200 g. Physical examinations were conducted prior to the experiment to ensure that the rats were healthy. The operated rats were divided into 3 groups: control group (topical normal saline three times a day, *n* = 6); AMS group (treated with topical AMS three times a day as instructions for use, *n* = 6); RGTA group (treated with topical RGTA once every other day, *n* = 6). Both tested topical agents were commercially available products and used according to the manufacturer's guidelines. AMS (EyeQ Amniotic Eye DropsⓇ), which contains morselized amniotic membrane, and Optixcare Eye EMSⓇ, containing RGTA as a replacement for heparan sulfate glycosaminoglycans, both provide an optimal healing environment for the repair of corneal tissue.

### 2.2 Animal model of deep corneal stromal wound

Before the surgical interventions, all subjects underwent thorough ophthalmic evaluations to ascertain the condition and well-being of their eyes. These evaluations included corneal fluorescein staining and assessments using a slit lamp biomicroscope. The lamellar keratectomy method employed to induce deep stromal ulcers was adapted from methodologies outlined in prior research (Ljubimov & Saghizadeh, 2015). All surgeries were only performed on one randomly selected eye of all rats. Preparation: The selected eye was prepared aseptically with a 50x diluted povidone-iodine solution. Anaesthesia: The induction and maintenance phase of anesthesia was achieved by intraperitoneal injection of ZoletilⓇ (25 mg / kg) (Virbac, Carros, France) and xylazine (10 mg/kg) (BalanzineⓇ, Elite Bio-Science inc., Taipei, Taiwan). Surgical procedures: Corneal microsurgery was performed under an operating microscope. To maintain uniformity in wound dimensions, a 3 mm biopsy punch (Disposable Biopsy PunchⓇ, Integra, Princeton, NJ, USA) was employed to delineate the surgical boundary on the corneal surface. This instrument was carefully rotated to demarcate the precise margin required. A sterile beaver blade #64 (Miniature Blade Round-Tip Sharp 1 SideⓇ, Surgistar, Vista, CA, USA) was used to create a stromal depth corneal incision. Lamellar keratectomy was performed using a corneal dissector and the beaver blade #64. Subsequently, corneal scissors were used to excise any redundant corneal tissue at wound periphery. Validation of surgical wound: Spectral domain-optical coherence tomography (SD-OCT) was used to guide and confirm the depth of the wound after the procedure. Pre-op and post-op medication: Meloxicam (2 mg/kg) (Melicam 7.5 mg, Synpac-Kingdom Pharmaceutical Co., LTD., Taoyuan, Taiwan) was orally administered at least 2 h prior to the surgical procedure. Additionally, meloxicam was administered once daily until the time of euthanasia, with a dosage of 2 mg/kg for the initial 2 days following surgery, and subsequently reduced to 1 mg/kg for the remaining duration. Topical tobramycin antibiotic (TobrexⓇ, Alcon, Fort Worth CA, USA) was applied to the operated eyes three times a day for seven days. The antibiotic was administered a minimum of 10 min prior to the applications of AMS or RGTA eyedrops. An E-collar was used to prevent secondary ocular injury by rubbing or scratching the affected eyes in the test rats. The E-collar was worn in the rats for the entire duration of the experiment to ensure consistent and reliable experimental results.

On postoperative day 3 and 7, rats were anesthetized for the corneal opacity grading, follow-up OCT examinations and subsequent euthanasia. After euthanasia, the eyes were collected for histopathological examinations. Intracardiac injection of potassium chloride (2 mmol/kg) was used as the euthanasia method. Intracardiac injection of potassium chloride, when administered under deep anesthesia, is considered a humane method of euthanasia, ensuring rapid cessation of cardiac activity with minimal distress to the animal. This approach is in strict adherence to the 'Guideline for the Care and Use of Laboratory Animals' as published by the Taiwan Ministry of Agriculture (Ministry of Agriculture publication no.1070043010A)

### 2.3 OCT analysis

SD-OCT was used to evaluate the cross-sectional images of the cornea. The progression of stromal healing was documented and evaluated (Famose, 2014). The thickness of the stromal layer of the central cornea was measured and documented using OCT and image analysis software (Heidelberg Eye Explorer, Heidelberg engineering, Franklin, MA, USA).

### 2.4 Histopathological and immunohistochemistry analysis

The globes of the rats were fixed in 10 % formalin after euthanasia. The specimens were then embedded in paraffin, cross-sectioned and stained with hematoxylin-eosin reagent.

Inflammatory cell counts within the corneal stroma per high-power field (HPF) were determined through manual calculation. The cornea was examined under HPF, and images were captured for each area to establish the average count of inflammatory cells within each HPF.

Immunohistochemical staining of all samples was performed using anti-alpha-smooth muscle actin (SMA) antibody (Agilent Technologies, Santa Clara, CA, USA). The staining protocol was performed essentially as described previously (Mudaliar et al., 2019). The number of myofibroblasts in the corneal stroma per HPF was calculated following staining for SMA. Surgical areas of the cornea were observed under HPF, and pictures of those areas were taken to calculate the average number of myofibroblasts under each HPF.

### 2.5 Corneal opacity grading

**Fig. 1:**
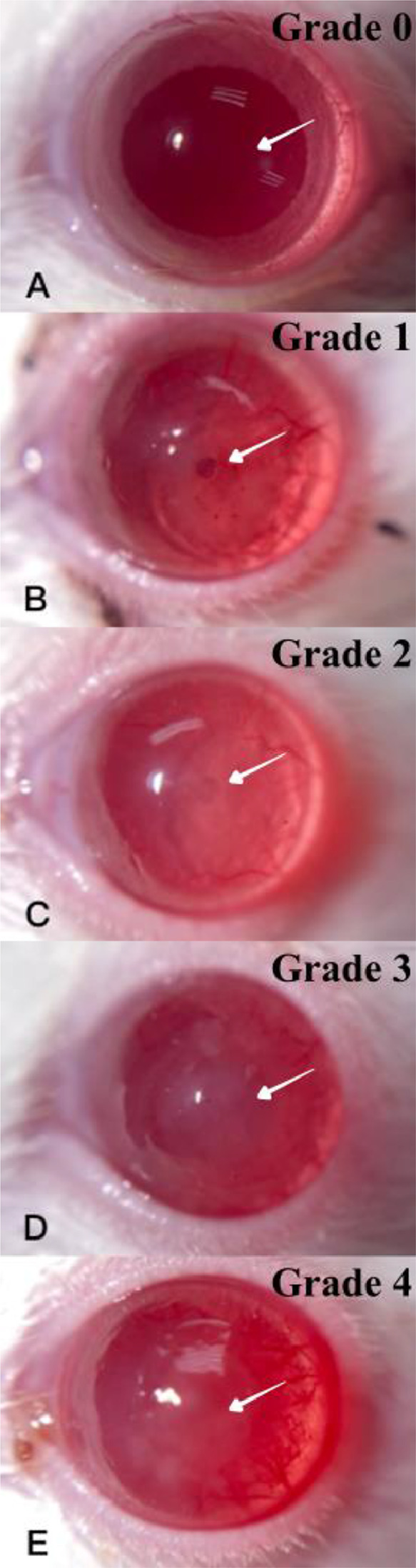
Gross pictures of different corneal opacity gradings. Corneal opacity grading was conducted at the corneal location indicated by the arrow. (A)corneal opacity grade 0; clear corneal transparency, (B)corneal opacity grade 1; mild opaque cornea, but the iris details were still visible, (C)corneal opacity grade 2; moderate corneal opacity, (D)corneal opacity grade 3; severe corneal opacity, but pupil margin was still visible, (E)corneal opacity grade 4; complete opaque cornea, iris was not visible.

Corneal opacity was evaluated and scored using a modified grading system adapted from a previous study (Yoon et al., 2008). Corneal opacity of the wounded area was graded from 0 to 4: 0, clear cornea; 1, mild stromal opacity and details of the iris visible; 2, moderate stromal opacity and pupillary margin still visible; 3, severe corneal opacity and can hardly detect the pupil; 4, opaque cornea with iris not visible. Pictures illustrating different corneal opacity gradings are featured in Fig. 1.

## 3 Statistical analysis

All data were analyzed using SPSS Statistics 27.0.1.0 for mac (IBM, Armonk, USA). Descriptive statistical analysis was performed, and the results were presented as mean ± standard deviation (SD) and graphs. One-way ANOVA was used to analyze variance between groups, and Dunnett's test was used for post hoc analysis. A *p*-value less than 0.05 was considered statistically significant.

## 4 Results

**Fig. 2:**
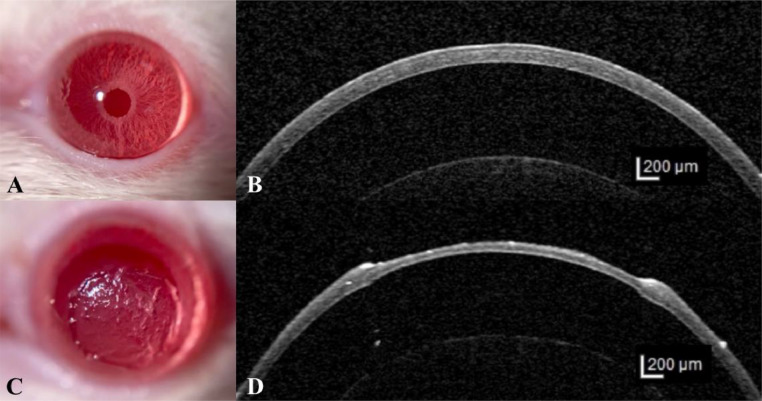
Gross and OCT images of cornea before and after surgery. (A) Gross image of normal rat cornea. (B) OCT image of normal rat cornea. (C) Gross image of rat cornea after the lamellar keratectomy. (D) OCT image of rat cornea after the lamellar keratectomy. Before the surgery, a transparent cornea allowed for clear visualization of intricate iris details. Following the surgery, a corneal defect was evident, as shown in both the gross image and OCT cross-sectional image. The scale bars for OCT images (B) and (D) measure 200 µm.

The preoperative evaluation of the cornea using fluorescein staining and slit lamp biomicroscope confirmed the intact and healthy condition of all tested corneas at day 0. Gross pictures and OCT images of cornea before and after surgery are presented in Fig. 2.

### 4.1 Corneal stromal thickness

The mean corneal stromal thickness for the three groups from day 0 to day 7 is detailed in Table 1. Prior to the lamellar keratectomy, there were no significant differences in corneal stromal thickness among the groups (*p*-value = 0.88). Post-surgical measurements showed no statistically significant differences in corneal stromal thickness between the groups (*p*-value = 0.63).

**Table 1:** The mean corneal stromal thickness ± standard deviation of 3 experimental groups.

	Before surgery	After surgery	Day 3	Day 7
Control group	84.66 ± 8.75 μm	50.06 ± 12.05 μm	51.18 ± 6.94 μm	55.27 ± 5.26 μm
AMS group	82.77 ± 6.13 μm	51.13 ± 3.04 μm	64.60 ± 4.79 μm	*70.38 ± 5.80 μm
RGTA group	84.94 ± 9.61 μm	54.43 ± 6.50 μm	59.59 ± 4.99 μm	*76.59 ± 6.80 μm

Significant differences were observed between both the AMS and RGTA groups in comparison to the control group on the seventh day after surgery (*p*-values were both < 0.05). Within the seven days of treatment, there was no significant increase in corneal stromal thickness observed in the control group. Conversely, both the AMS and RGTA groups exhibited a notable increase in corneal stromal thickness during the same period.

#### 4.1.1 Postoperative day 3

There were no significant differences in stromal thickness between groups (*p*-value= 0.11).

#### 4.1.2 Postoperative day 7

Significant differences were observed in the AMS and RGTA groups when compared to the control group (*p*-value were 0.038 and 0.009, respectively).

### 4.2 Analysis of OCT images

#### 4.2.1 Postoperative day 3

Upon OCT evaluation, a significant increase in epithelial tissue thickness was observed in the damaged areas compared to the epithelial layer of the undamaged regions. Moreover, the OCT images revealed increased reflectivity within both the epithelial and stromal layers across all groups, distinguishing the injured corneal tissue from the healthy tissue.

#### 4.2.2 Postoperative day 7

In the RGTA group, the stromal tissue displayed elevated levels of hyperreflectivity when compared to both the untreated control and the AMS groups.

### 4.3 Histopathology and immunohistochemistry analysis

#### 4.3.1 Postoperative day 3

Within the surgically modified region, there was a clear presence of hypertrophy and hyperplasia of the corneal epithelium. Compared to the control and AMS groups, which showed fibroblasts that were more loosely organized, the RGTA group demonstrated a comparatively higher density of fibroblasts.

Moderate infiltration of neutrophils and a small number of macrophages were detected in all treatment groups on the third day following surgery. Most of the inflammatory cells were concentrated within the surgical zone, predominantly situated in the stromal layers that are in close proximity to the corneal epithelium.

Myofibroblasts were present in all groups, as evidenced by positive staining for SMA in immunohistochemical analysis. The control and AMS groups had a limited number of SMA-positive cells, mostly concentrated near the epithelial layer. However, the RGTA group showed a notably larger population of myofibroblasts distributed throughout the stromal tissue in comparison to the other groups.

#### 4.3.2 Postoperative day 7

**Fig. 3:**
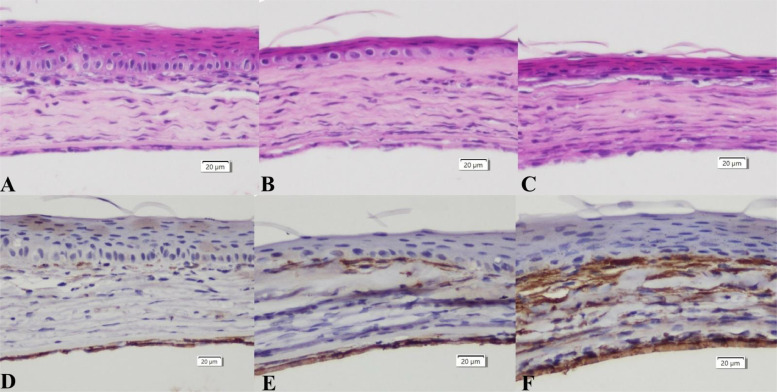
Histopathological images and SMA stains of cornea at post-operative day 7. Spindle-shaped cells could be observed under H.E staining in all experimental groups. And smooth muscle actin (SMA)-positive cells were observed in all groups. (A) H.E staining of the control eye, (B) H.E staining of the AMS group, (C) H.E staining of the RGTA group, (D) SMA stain of the control eye, (E) SMA stain of the AMS group, (F) SMA stain of the RGTA group. Intense SMA-positive cells infiltration could be found in the RGTA group. The scale bar for all images measures 20 µm.

Histopathological images of the cornea in all treatment groups on day 7 are shown in Fig. 3. While hyperplasia of the corneal epithelial cells was still noticeable within the surgical area, its extent was less prominent.

In both the control and AMS groups, myofibroblasts were detected in a multifocal distribution within the injured stromal regions, with the majority being situated close to the epithelial layer. By comparison, the RGTA groups showed a significantly denser infiltration of myofibroblasts throughout the stromal tissue.

### 4.4 Myofibroblast counts

Myofibroblast counts per HPF on post-operative day 3 and 7 of all groups are shown in Table 2.

**Table 2:** The mean myofibroblast counts ± standard deviation per high-power field (HPF).

	Day 3	Day 7
Control group	2.54 ± 0.74 cells/HPF	3.49 ± 3.36 cells/HPF
AMS group	1.28 ± 0.92 cells/HPF	5.70 ± 0.30 cells/HPF
RGTA group	6.92 ± 4.49 cells/HPF	*16.95 ± 6.04 cells/HPF

Myofibroblasts were found in all four groups at both post-operative day 3 and day 7. The myofibroblast counts per HPF in the RGTA group was significantly higher than the control group. (*p-value was 0.01).

#### 4.4.1 Postoperative day 3

Statistical analysis indicated that there were no significant differences in the myofibroblast counts per HPF between the groups (*p*-value = 0.09).

#### 4.4.2 Postoperative day 7

A significant difference was noted in the myofibroblast counts for the RGTA group in comparison to the control group (*p*-value was 0.011). On the other hand, the AMS group did not show a significant difference in myofibroblast counts when compared to the control group (*p*-value = 0.88).

### 4.5 Inflammatory cells count

Inflammatory cells count per HPF on post-operative day 3 and 7 of all groups are shown in Table 3.

**Table 3:** The mean inflammatory cell counts ± standard deviation per high-power field (HPF).

	Day 3	Day 7
Control group	10.98 ± 10.19 cells/HPF	3.36 ± 0.77 cells/HPF
AMS group	10.11 ± 2.52 cells/HPF	1.81 ± 0.26 cells/HPF
RGTA group	13.41 ± 11.79 cells/HPF	4.28 ± 1.38 cells/HPF

Intense inflammatory responses were observed in all groups on post-operative day 3, with neutrophils and macrophages being the predominant inflammatory cells. There was no significant difference in the count of inflammatory cells per HPF between post-operative day 3 and day 7.

#### 4.5.1 Postoperative day 3

Statistical analysis revealed no significant differences between the groups (*p*-value= 0.90).

#### 4.5.2 Postoperative day 7

Statistical analysis revealed no significant differences between the groups (*p*-value= 0.10).

### 4.6 Corneal opacity grading

#### 4.6.1 Postoperative day 3

On day 3, no significant differences were noted in the corneal opacity gradings between the control, AMS, and RGTA groups (*p*-value = 0.43). The mean opacity grades were 2 ± 0.63 for the control group, 2.17 ± 1.17 for the AMS group, and 2.33 ± 1.21 for the RGTA group.

#### 4.6.2 Postoperative day 7

A significant difference in corneal opacity was detected among the groups, with the RGTA group showing significantly higher corneal opacity grades in comparison to the control group (*p*-value = 0.009). The average corneal opacity grades for the control, AMS, and RGTA groups were 1.00 ± 0.00, 1.33 ± 0.57, and 2.67 ± 0.57, respectively.

## 5 Discussion

Deep stromal ulcers, unlike superficial corneal ulcers which often improve with just medical treatment, typically necessitate surgical intervention in the field of veterinary eye care. Surgical procedures are key in promoting the healing process of deep stromal ulcers and in mitigating potential severe complications like globe rupture or melting changes. A wound is categorized as a "deep stromal wound" when its depth extends to 50 % or greater than the original thickness of the cornea (Gelatt et al., 2021). In this rat model, the baseline average corneal thickness was 116.40 ± 1.94 μm. After surgery, the average corneal thickness was reduced to 51.87 ± 2.27 μm, which is below 50 % of the initial thickness, thus qualifying as a deep stromal ulcer. The size and depth of the corneal wounds in all the test subjects were maintained as uniform as possible, as confirmed by OCT.

The corneal stromal healing process can be spanning weeks to months in small animals (Maggs et al., 2017). In this rat model of corneal stromal wounds, the fluorescein staining of corneal lesions turned negative from post-operative day 3 onwards, indicating the healed epithelial layer of induced corneal wound since day 3. Moreover, no significant differences were observed in the corneal epithelial healing process with the use of both AMS and RGTA. The recovery of the stromal wounds, as assessed by OCT, was observed between days 14 and 21 (data not shown) in the pilot studies. The primary and significant healing phase occurred in the initial week following surgery, after which the healing process notably slowed down and became minimal past the seventh post-operative day in this model. The measurement data for corneal stromal thickness and corneal opacity grading remained relatively consistent without significant changes from post-operative day 7 to day 21. Consequently, our experimental design concentrated on the critical early phase of stromal healing in this rat model, a decision informed by observations from pilot studies.

OCT serves as a valuable tool for obtaining high-resolution cross-sectional tomographic images of tissue, functioning similar to an optical biopsy. The elevated stromal signal intensity observed in OCT images indicates the presence of an active stromal healing process, including phenomena such as inflammatory cell infiltration and corneal fibrosis, as demonstrated in previous studies (Famose, 2014; Zheng et al., 2016). In our study, both the AMS and RGTA groups exhibited a significant increase in corneal stromal thickness over the seven-day treatment period, as assessed by OCT examination. In the control group, there was no substantial increase in corneal stromal thickness during the first three days, with only a slight increase noted on the seventh day of treatment. The increase in stromal thickness in both AMS and RGTA groups was evident from day three and continued steadily, implying that both treatment groups may expedite corneal stroma healing relative to the control group.

On day seven of treatment, the RGTA group showed a significant rise in signal intensity of stromal tissue, indicative of substantial cellular infiltration or pronounced corneal fibrosis in this area. This finding from OCT corresponds with observed increased corneal opacity and a marked rise in myofibroblast infiltration in the RGTA group. This observation implies that RGTA treatment resulted in a substantial escalation of myofibroblast infiltration during the initial phase of the corneal stromal healing process.

For patients suffering from deep corneal ulcers, the optimal method to restore corneal structural stability and preserve optimal transparency is through corneal surgical procedures, such as corneoconjunctival transposition or autologous corneal grafting (Gogova et al., 2020; Jaksz et al., 2021). However, for patients who are not candidates for general anesthesia, or for those facing financial constraints, a medical treatment that can similarly promote the healing process of the stromal layer presents a valuable option. In previous study, AMS had been shown to reduce corneal opacity and promote epithelial healing on alkali burn rat model (Choi et al., 2011). However, in our animal model, we observed that AMS improved stromal healing without a significant effect on corneal opacity. It is possible that the differing results regarding corneal opacity could be attributed to the variations in the animal models used. While RGTA significantly promotes corneal stromal healing, it has also been linked to a significant increase in myofibroblast infiltration and corneal fibrosis during stromal wound healing process in this study.

Deep corneal ulcers in small animals are commonly associated with infections, which create an environment in the cornea that tends to promote the activity of proteinases over proteinase inhibitors (Gelatt et al., 2021). As a result, conditions such as stromal malacia or melting ulcers are commonly seen. The products we utilized in our research are believed to have properties that could counteract the effects of exogenous proteases released during infections and have a beneficial impact on infected corneal ulcers. In previous research, the Amniotic membrane demonstrates anti-bacterial properties (Mamede et al., 2012), and RGTA has been shown to be resistant to enzymatic degradation (Barritault et al., 2017). Nevertheless, in this study's deep corneal ulcer model, no infection was induced in the eyes that underwent surgery. Therefore, we couldn't establish or compare the antibacterial effectiveness of these products against a control group. More research and clinical trials are needed to evaluate their efficacy in combating infection and enzymatic degradation in infected corneal ulcers.

## 6 Conclusion

The study found that both topical AMS and RGTA treatments promoted the corneal stromal wound healing process. These outcomes demonstrated the potential benefits of AMS and RGTA for the clinical treatment of deep corneal ulcers. The highest levels of stromal wound remodeling such as myofibroblast infiltration and wound defect recovery were noted in the RGTA treated group based on the findings. The outcomes of this study indicated that both AMS and RGTA may be beneficial in treating deep corneal ulcers in small animal practice. Further research is necessary to investigate the long-term effects and mechanism of using topical AMS and RGTA on treating deep corneal ulcer in clinical practice.

## Funding

This research did not receive any specific grant from funding agencies in the public, commercial, or not-for-profit sectors.

## Ethical statement

All protocols in this study were approved by the Institutional Animal Care and Use Committee (IACUC) NTU, Ethic research center, National Taiwan University, Taipei, Taiwan **(IACUC permit number: NTU-110-EL-00082)**, in compliance with the Guideline for the Care and Use of Laboratory Animals published by the Taiwan Ministry of Agriculture (Ministry of Agriculture publication no.1070043010A).

## CRediT authorship contribution statement

**Hao Lee:** Data curation, Formal analysis, Investigation, Methodology, Writing – original draft. **Wei-Hsiang Huang:** Investigation, Validation. **Yi-Chen Sun:** Investigation, Validation. **Chung-Tien Lin:** Conceptualization, Supervision, Resources, Writing – review & editing.

## Declaration of competing interest

The authors declare that they have no known competing financial interests or personal relationships that could have appeared to influence the work reported in this paper.
